# Evacuation of a spontaneous massive spinal epidural hematoma by a minimally invasive surgical technique: a case report

**DOI:** 10.11604/pamj.2022.43.55.36552

**Published:** 2022-10-04

**Authors:** Mouhssine Assamadi, Lamia Benantar, Hajar Hamadi, Bader Drai, Omar Ksiks, Tarik Belokda, Elmehdi Hamidi, Khalid Aniba

**Affiliations:** 1 Department of Neurosurgery, Ibn Tofail Hospital, Mohammed VI^th^ University Hospital, Faculty of Medicine and Pharmacy in Marrakech, Cadi Ayyad University, Marrakech, Morocco

**Keywords:** Spontaneous, thoracolumbar, anticoagulant therapy, surgical evacuation, case report

## Abstract

Spontaneous spinal epidural hematomas are a rare consequence of long-term anticoagulant therapy. Their physiopathology remains poorly understood. This pathology carries a significant risk of morbidity. The purpose of this article is to report a case of a massive spontaneous spinal epidural hematoma extending on multiple levels, detailing the surgical technique used in its evacuation described for the first time in literature. This is a case report of an 80-year-old patient on anticoagulant therapy with a thoracolumbar spontaneous spinal epidural hematoma extending from T1 to L1 vertebrae. We share the clinical and radiological presentations, the surgical treatment, outcome and follow-up. The diagnosis of spontaneous spinal epidural hematoma has to be considered in patients with acute brutal onset radiculo-medullary compression. Medullary magnetic resonance imaging (MRI) remains the exam of choice. Medical and surgical treatment must be started immediately after the diagnosis is confirmed. The prognosis remains poor despite a proper management, with debilitating complications.

## Introduction

Bleeding disorders due to long-term anticoagulant therapy are one of the rare causes of spontaneous spinal epidural hematomas (SSEH). Regular surveillance of the international normalized ratio (INR) isn´t always helpful in the prevention of these complications. The physiopathology and origin of the hematoma (venous or arterial) remain poorly understood [1-4]. Though the diagnosis of SSEH is easily thought of in cases of rapid onset radiculo-medullary compression then rapidly confirmed with magnetic resonance imaging (MRI), and even with fast proper management, this pathology carries a significant risk of morbidity with extremely debilitating neurological sequelae [2,3].

In literature, the majority of authors describe cases of SSEH involving 2 to 5 vertebral segments allowing for an easier surgical evacuation. The latter becomes more challenging when dealing with extensive hematomas due to higher risks of iatrogenicity [3-10]. In this article, we report the case of a massive thoracolumbar SSEH extending from T1 to L1 vertebrae revealed by an acute onset paraplegia in a patient on anticoagulant therapy. We detail the surgical technique used in its evacuation while avoiding extensive laminectomy. The before mentioned technique hasn´t been described nor mentioned -as far as our research scope extends- anywhere in the current articles of literature, making this report that much more pertinent to the management of this considerably rare pathology.

## Patient and observation

**Patient information:** the patient, is an 80-year-old man with personal history of diabetes mellitus on insulin, hypertensive cardiopathy treated with diuretics, and cardiac arrhythmia due to atrial fibrillation under vitamin K antagonists (Warfarin) for 3 years with a daily dose of 4 mg. He presented with excruciating spontaneous back and interscapular pain, complicated with progressive motor deficit beginning in the right lower extremity then in both lower extremities, and acute urinary retention 3 hours after onset.

**Clinical findings:** clinical examination (12 hours after the beginning of symptoms) showed a flaccid paraplegia (manual muscle testing = MMT score of 0/5 in all the muscle groups of both lower extremities), total anesthesia with a T4 sensory level (corresponding to a grade A on Frankel scale), areflexia, bilateral Babinski sign, a full bladder and anal incontinence on rectal examination.

**Figure 1:**
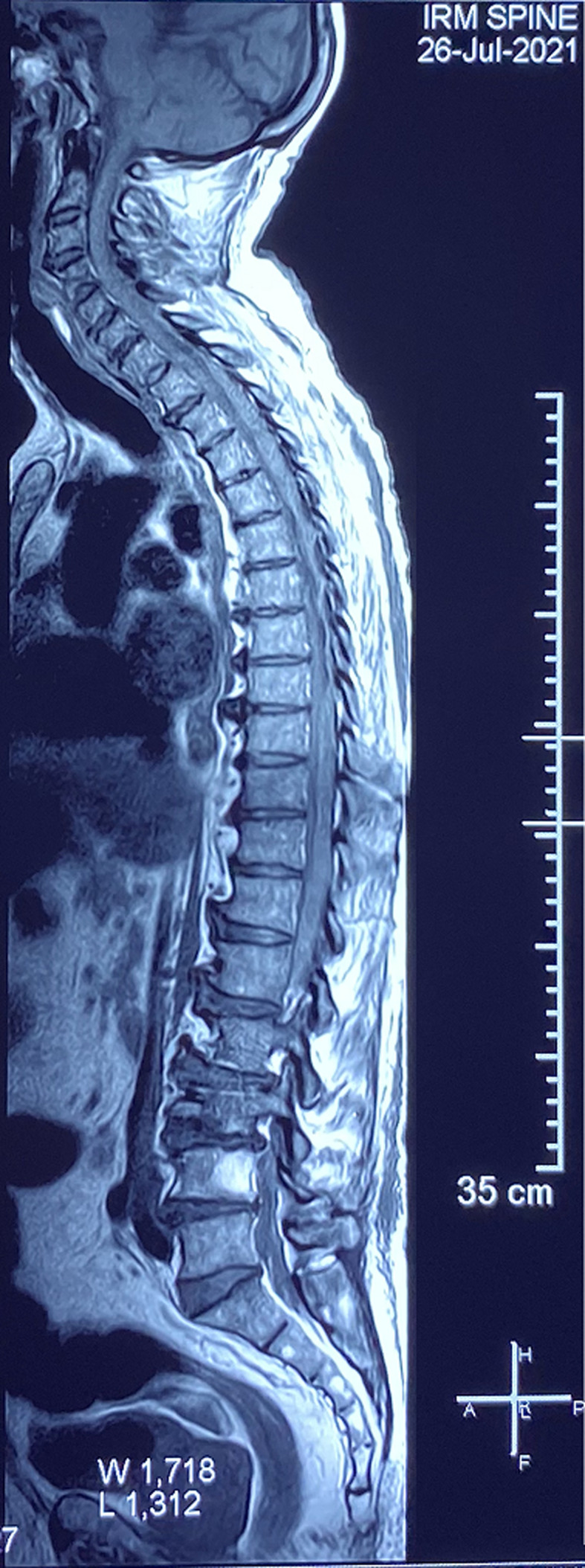
medullary MRI in T1 weighted sequence sagittal plane; medullary MRI sagittal plane showing a well circumscribed epidural lesion in isosignal T1 extending from D1 to L1

**Figure 2:**
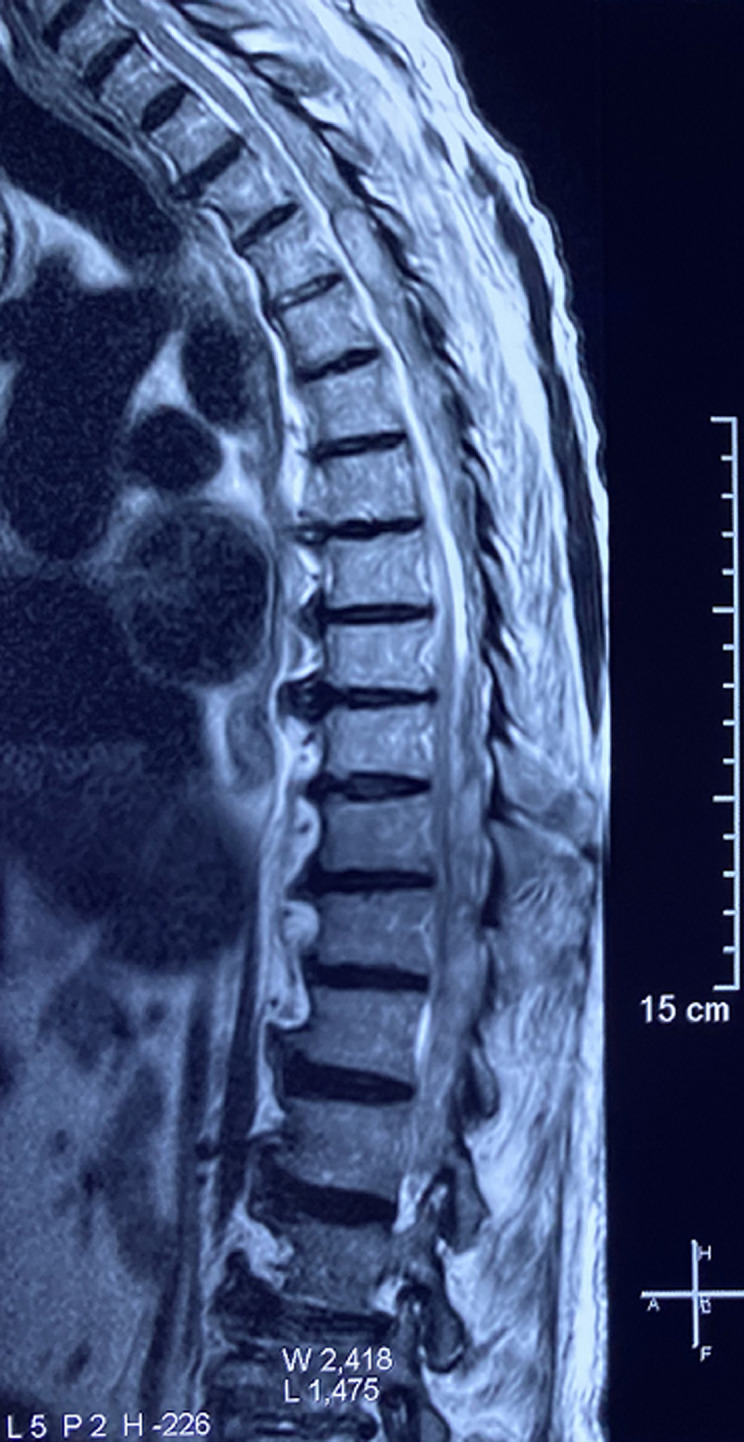
medullary MRI in T2 weighted sequence sagittal plane; medullary MRI sagittal plane showing the same well circumscribed epidural lesion in heterogenous hypersignal T2 extending from D1 to L1

**Figure 3:**
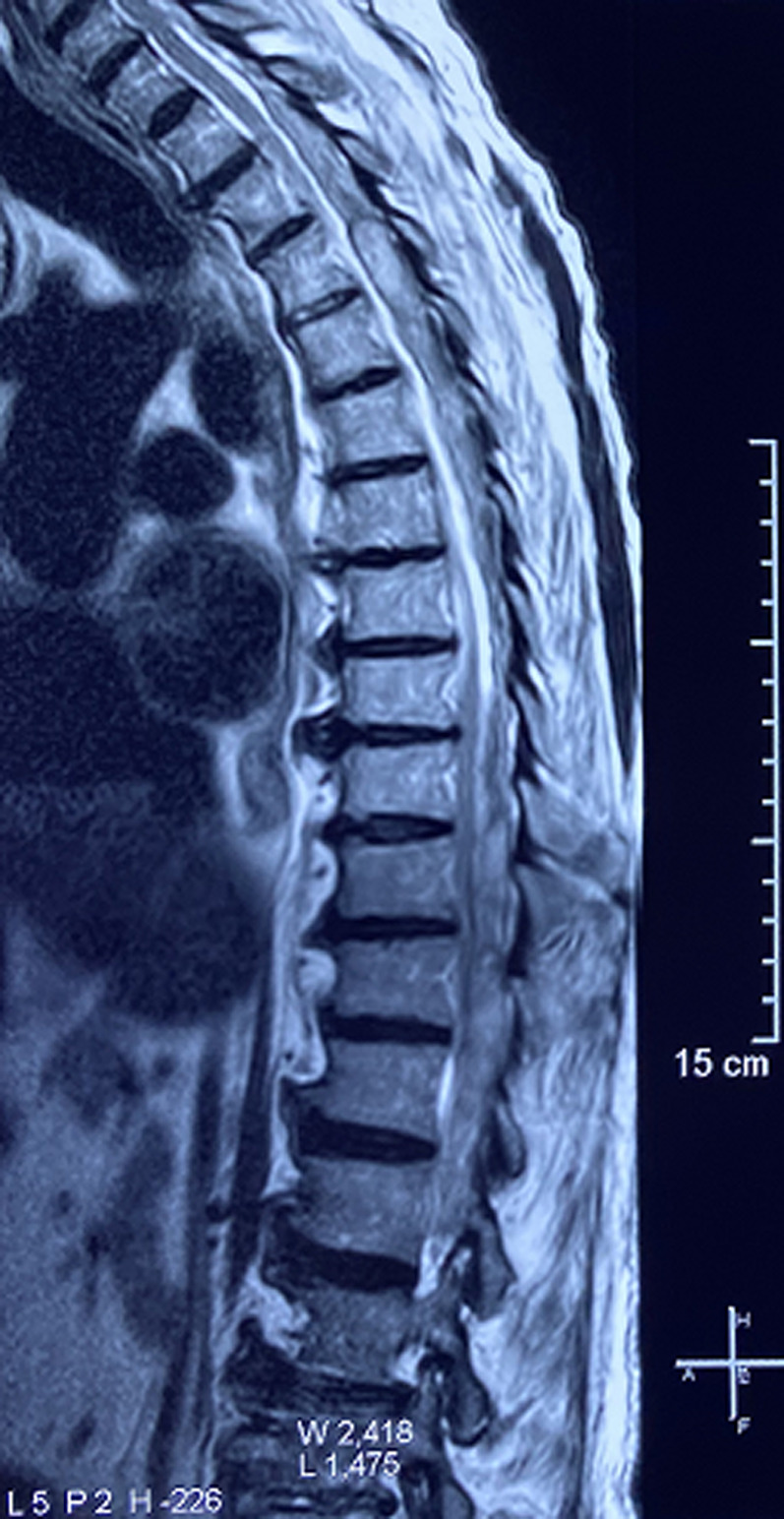
medullary MRI in gradient echo sequence sagittal plane; medullary MRI sagittal plane showing the epidural lesion in hypersignal gradient echo with a peripheral halo on hyposignal

**Figure 4:**
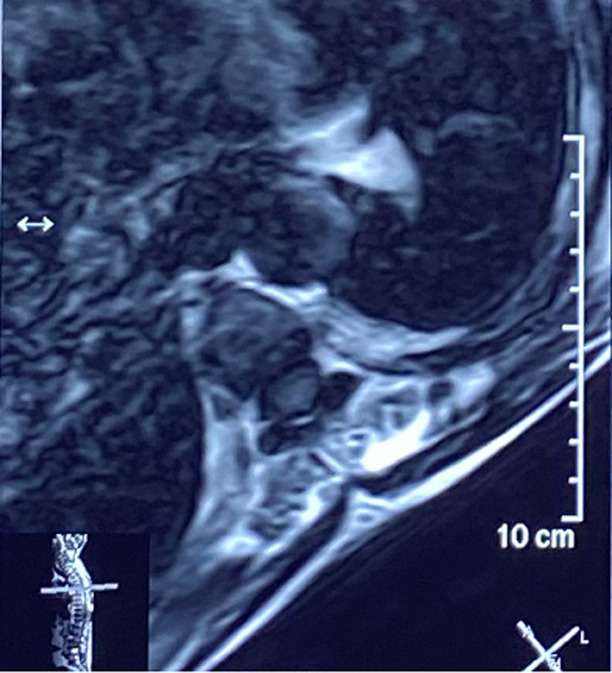
medullary MRI in T1 weighted sequence axial plane; medullary MRI axial plane showing the epidural lesion in Isosignal T1 which appears to be slightly deviated to the right

**Figure 5:**
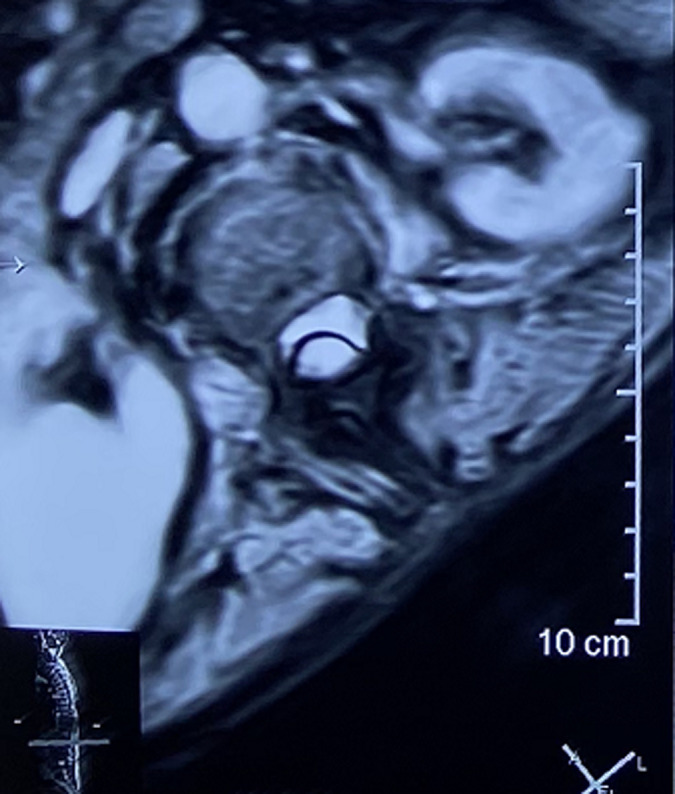
medullary MRI in T2 weighted sequence axial plane; medullary MRI axial plane showing the well circumscribed epidural lesion in heterogenous hypersignal T2 slightly deviated to the right, this image is highly evocative of an acute spinal epidural hematoma

**Diagnostic assessment:** a medullary MRI was performed promptly showing an epidural hematoma extending from T1 to L1 vertebrae, slightly lateralized to the right, compressing the spinal cord at the thoracic level as well as the conus medullaris (Figure 1, Figure 2, Figure 3, Figure 4, Figure 5). The MRI also showed a peritoneal effusion on the abdominal slices. A complete blood test showed a prothrombin time (PT) inferior to 10%, and an INR value superior to 10, with a normochromic normocytic anemia of 6.3g/dL and a platelet count of 220000/mm^3^.

**Therapeutic interventions:** a urinary catheter was immediately placed before further investigations. Once the diagnosis of SSEH secondary to a Warfarin overdose was confirmed emergency treatment was instituted, starting with the administration of an oral dose of 10 mg of vitamin K to counter the effects of Warfarin, as well as the intravenous administration of prothrombin complex concentrate with a dose of 25UI/kg. INR control value an hour later was 1.53 and PT value was 69%. Following the blood test results (which were acceptable for surgical treatment), the patient was taken to the operation room for emergency evacuation of the hematoma. The surgical procedure consisted of a laminectomy at T6-T7 levels, followed by the evacuation of the hematoma at the laminectomy level by saline irrigation and aspiration. To evacuate the hematoma at the other levels (T1-T5 and T8-L1), a flexible suction catheter size 8 was introduced in the epidural space. At the end of the procedure, total evacuation of the hematoma was achieved followed by a satisfying hemostasis. The surgical incision was closed leaving a Redon close suction drain in the subfascial plane.

**Follow-up and outcome of interventions:** post-operative (PO) follow-up was uneventful, the drain was removed at PO day 2 and drained out 230 ml of hematic fluid. The patient was discharged at PO day 5 with a urinary catheter and an anticoagulant therapy (low molecular weight heparin relayed with Warfarin) prescribed and controlled by his cardiologist. Physical and sphincter rehabilitation were started on PO day 2 and continued to date (6 months PO). On his most recent follow-up, the patient hasn´t fully recovered complete motor function and sphincter control. He was able to stand with help, MMT score was 3/5 in the right lower extremity and 4/5 on the left (corresponding to a grade B on Frankel scale), and the use of a urinary catheter was still necessary. Control medullary MRI was not performed due patient reticence regarding the coast and lack of means.

**Patient perspective:** the patient was made aware of the severity of the functional prognosis concerning his pathology due to the delayed consultation and the extend of the hematoma. However, despite the lack of full recovery of motor and sphincter functions, the patient was satisfied of the outcome of his surgery at 6 months PO follow-up.

**Informed consent:** written informed consent was obtained from the patient for participation in our study.

## Discussion

Spontaneous spinal epidural hematomas (SSEH) is defined as the presence of a hematoma in the spinal epidural space in the absence of traumatic and iatrogenic causes (lumbar punction, spine surgery) [2,7,10]. Some authors include in this definition hematomas due to a coagulopathy, a vascular malformation or tumors [3,7,10,11], while others include idiopathic hematomas only [5,6,12]. SSEH was first described by Jackson in 1869 as a post-mortem discovery [13], while the first surgical evacuation via a posterior approach was attempted at New York´s Presbyterian Hospital by Ver Brugghen in 1943 [14]. During that time the diagnosis was difficult leading to underdiagnosed cases of SSEH. Despite the advent of better imaging techniques such as MRI, this pathology remains rare representing less than 1% of all epidural lesions [2,6,15] with an estimated incidence of 1 case per 1 million per year [2,6,7,10,15]. Though many etiologies can lead to SSEH, the main cause remains coagulopathies due to anticoagulant treatment comprising 17 to 30% of all causes [2,4,6,10].

Despite being an uncommon pathology, rarely encountered during a neurosurgeon´s career, its morbidity and mortality are significantly high [2,3], and has to be considered as a probable diagnosis in old patients on anticoagulants presenting with a brutal onset radiculo-medullary compression [2].

The clinical presentation begins with acute intense cervicalgia with interscapular pain, and depending on the location of the hematoma; dorsalgia or lombalgia may be present. Minutes to hours later, rapidly progressive incomplete motor deficit is seen, to which sphincter deficits are often associated [2-4,10,15]. Certain clinical forms can be misleading, mimicking an expelled herniated disc, a spondylodiscitis, an epiduritis due to a tumor or infection, but the rapid deterioration of initial symptoms is highly suggestive of a hemorrhagic vascular pathology [7,15].

Magnetic resonance imaging (MRI), is the imaging of choice, as it confirms the diagnosis of SSEH and rules out other differential diagnosis. It allows for a visualization of the location and extent of the lesion and its limits. The difficulty lies in the interpretation of the images since the signal of the hematoma varies with time (depending on the level of oxygenation of hemoglobin and the state of red blood cells membranes), which imposes a careful reading of the images taking into account the evolution period of the clinical presentation [2-4, 6, 7,10,11,16].

A surgical evacuation is indicated each time a neurological deficit is present. The surgical procedure consists of a laminectomy followed by an evacuation of the hematoma and careful hemostasis [2-6,10]. The majority of SSEH cases found in literature describe limited hematomas, extending on 2 to 5 levels making a direct surgical approach acceptable [3-10]. The particularity of our case is the large extent of the hematoma including 13 levels from T1 to L1 vertebra, which raises the issue of iatrogenic complications. A laminectomy of 13 levels means [16-18]: a large surgical incision: high hemorrhagic risk and consumption of clotting factors; a prolonged operating time: significantly high infection rate; an extensive surgical detachment of the paravertebral muscles: post-operative and long-term residual pain, weakness of the paravertebral musculature; an extensive laminectomy: a high risk of a dural breach or a radiculo-medullary lesion, as well as a post-operative biomechanical instability and spinal deformity; a long recovery period with higher risks of complications related to prolonged immobility.

To reduce the risk of iatrogenicity, we realized a surgical approach with a limited incision to the middle 1/3 of the lesion extending on 2 levels from T6 to T7. A T6-T7 laminectomy was performed followed by the evacuation of the hematoma at the corresponding level. The evacuation of the hematoma at the above and below levels, was aided using a flexible aspiration catheter size 8. The surgical technic consisted of a careful and slow introduction of the catheter, then hydrodissection of the hematoma was done using an injection of small quantities of physiological 0.9% saline solution to avoid further compression, followed by gentle suction. This procedure was repeated progressively at each level until complete evacuation is obtained made evident by the evacuation of clear saline solution free of residue, which is also a sign of a satisfying hemostasis. The closure of the surgical incision was done plane by plane leaving in place a subfascial Redon close suction drain.

Despite a good surgical decompression, the clinical evolution and recovery of our patient was not satisfying due to the presence of poor prognosis factors, including: age of the patient, severe clinical presentation with sphincter deficit, the extent of the lesion as well as the period between the initial presentation and surgical treatment (superior to 12 hours) [2-8].

## Conclusion

Spontaneous spinal epidural hematomas (SSEH) is a rare pathology with a significantly high morbidity and mortality. The diagnosis has to be considered in patients presenting with acute brutal onset radiculo-medullary compression. Medullary MRI is the exam of choice to confirm the diagnosis. The treatment is both medical and surgical, consisting of bleeding and coagulopathy management, allowing for an emergency evacuation and decompression of the spinal cord using a minimally invasive technique, thus avoiding iatrogenic complications that could further compromise the prognosis.
